# Use of Cultured Cells to Study Alcohol Metabolism

**Published:** 2006

**Authors:** Dahn L. Clemens

**Affiliations:** Dahn L. Clemens, Ph.D., is associate professor in the Department of Internal Medicine, University of Nebraska Medical Center and Veterans Affairs Medical Center, Omaha, Nebraska

**Keywords:** Abuse, and dependence, alcoholic liver disease, alcoholic fatty liver, ethanol metabolism, ethanol metabolite, cultured cells, recombinant cell lines, hepatocyte, alcohol dehydrogenase (ADH), acetaldehyde, cytochrome P450 2E1

## Abstract

The use of cells grown in the laboratory (i.e., cultured cells) in alcohol research has many advantages. Among these are the ability to investigate individual metabolic pathways, the ability to precisely control exposure to ethanol and its metabolites in the absence of confounding variables, and the uniformity of genetically identical (i.e., clonal) cell lines. Additionally, because of the cost and relative ease of culturing large quantities of cells, many more experimental replicas may be performed to confirm findings. As described in this article, the use of cultured cells has contributed greatly to the understanding of the mechanisms by which alcohol metabolism affects cells and ultimately results in alcoholic liver disease.

The association between alcohol abuse and liver disease has been recognized for centuries. Over time, we have gained a tremendous amount of knowledge regarding the effects of ethanol, the alcohol normally found in alcoholic beverages, on the liver. Unfortunately, many of the molecular mechanisms by which ethanol causes liver damage remain unclear. This article briefly reviews the contributions that the use of cells grown in the laboratory (i.e., cultured cells) have made to the understanding of the mechanisms by which alcohol metabolism affects cells, ultimately resulting in alcoholic liver disease.

The most abundant cell type in the liver, the hepatocytes, metabolize the vast majority of ingested alcohol; thus, ethanol metabolism is thought to be primarily responsible for ethanol-induced liver damage. Hepatocytes metabolize ethanol through two major metabolic pathways. The primary pathway is mediated by alcohol dehydrogenase, an enzyme located in the cytoplasm of hepatocytes. The cytoplasm is the semifluid part of the cell located between the cell membrane and the nucleus. The other pathway is mediated by cytochrome P450 2E1, an enzyme bound to the network of membranes within the cell known as the endoplasmic reticulum.

Ethanol metabolism results in a number of biochemical changes, including the production of the toxic byproduct acetaldehyde, the production of cell-damaging reactive oxygen and nitrogen species (i.e., molecules that are highly reactive because of the presence of unpaired electrons), a deficiency of oxygen in the tissues (i.e., hypoxia), and an increased ratio of the reduced form of the coenzyme nicotinamide adenine dinucleotide (i.e., NADH) to the oxidized form of nicotinamide adenine dinucleotide (i.e., NAD^+^).^1^ All of these biochemical changes have been proposed to contribute to hepatocyte injury and liver disease, although no single change can account for all of the effects of ethanol metabolism on the liver. In fact, in many cases two or more biochemical changes may act in concert to produce specific effects.

The molecular mechanisms of ethanol-induced liver damage have been difficult to determine because these biochemical changes occur simultaneously. Therefore, using animal models, it has been extremely difficult to establish a causeand-effect relationship between specific biochemical changes and specific pathologic changes. Additionally, freshly isolated hepatocytes rapidly regress to simpler, unspecialized cells (i.e., dedifferentiate) in culture, thereby losing the ability to carry out many hepatocyte-specific functions, including the ability to metabolize ethanol. Because of this, isolated hepatocytes have been of limited utility in defining the molecular mechanisms by which ethanol metabolism affects cells. To circumvent this problem, researchers have used recombinant DNA technology (i.e., combining DNA molecules that are not found together naturally) to create a number of recombinant cell lines of hepatic origin that express alcohol dehydrogenase, cytochrome P450 2E1, or both. Recombinant cell lines have enabled detailed investigation of the effects of ethanol metabolism in an environment where it is possible to directly evaluate the contribution of specific biochemical changes.

The effects of ethanol metabolism on the liver can broadly be placed into three categories: (1) effects on hepatocyte functions, (2) effects on hepatocyte viability, and (3) effects on hepatocyte replication. Cell culture models have been used to investigate these effects in detail, providing insight into the molecular mechanisms of ethanol-induced liver damage.

## Effects of Ethanol Metabolism on Hepatocyte Functions

The liver is responsible for the removal of many toxic substances from the blood, as well as the synthesis and secretion of many compounds. The most abundant cells in the liver, the hepatocytes, primarily carry out these functions. It has long been known that one of the biological consequences of chronic ethanol consumption is enlargement of the liver (i.e., hepatomegaly). The mechanism of this increase in liver volume is likely complicated and multifactoral, but two factors that may contribute to this change are impaired movement of proteins around a cell (i.e., protein trafficking) and impaired protein degradation.

To more closely investigate the effects of ethanol metabolism on protein trafficking, we stably introduced DNA into the well-differentiated liver cancer (i.e., hepatoma) cell line, Hep G2, so that the cells would express alcohol dehydrogenase (Clemens et al. 1995). Culturing these cells in media containing ethanol, we investigated the effects of ethanol metabolism on the ability of cells to take up material from the environment. This process is mediated by specific proteins on the surface of cells (i.e., receptors) that recognize and bind to specific substances or chemical messengers outside of the cell. This process is known as receptor-mediated endocytosis. Using the hepatocyte-specific asialoglycoprotein receptor and the substance that specifically binds to it (i.e., ligand), asialoorosomucoid, as a model, we showed that ethanol metabolism not only decreased the binding of the ligand to the receptor but also impaired internalization and degradation of the ligand. Subsequent experiments, in which the metabolism of ethanol was chemically inhibited, completely ameliorated these ethanol-mediated impairments, thus conclusively demonstrating that ethanol metabolism was required for these impairments. Furthermore, we found that increasing the exposure of the cells to the metabolic byproduct of ethanol metabolism, acetaldehyde, increased the severity of the impairments to receptor-mediated endocytosis. These results led us to suggest that acetaldehyde has a critical role in ethanol metabolism–mediated impaired receptor-mediated endocytosis.

In many cells that are organized into tissues, the cell membrane is not uniform (i.e., exhibits membrane polarity); this is because different domains of the cell membrane have different functions. Hepatocytes are no exception. Their basal lateral domain of the cell membrane is exposed to the liver sinusoid, or the blood supply, as well as the narrow intercellular space between adjacent hepatocytes, and their apical domain of the cell membrane is exposed to the tube or space between liver cells that collects bile from the cell (i.e., the bile canaliculus) (see Figure). Many cells, including hepatocytes, lose their polarity in culture. To circumvent this problem, Shanks and colleagues (1994) created a polar cell line with hepatic characteristics. The resulting WIF-B cell line expressed both alcohol dehydrogenase and cytochrome P450 2E1, maintained polarity, and formed functional bile canaliculi (Shanks et al. 1994). These cells have been used to investigate the effects of ethanol metabolism on the functions of protein structures that give the cell its shape and facilitate the movement of proteins and organelles throughout the cell (i.e., microtubules). Microtubules are required for a number of critical cellular functions, including organized intracellular protein trafficking. Culturing WIF-B cells in the presence of ethanol for 3 days reduced microtubule polymerization. Morphologically, the microtubules in cells that were cultured in the presence of ethanol appeared gnarled and shorter, characteristics commonly associated with stable microtubules. Analysis revealed that the major component of microtubules, α-tubulin, isolated from ethanol-treated cells was more highly altered by the introduction of an acetyl group (i.e., acetylated) compared with cells cultured in the absence of ethanol. Using specific biochemical inhibitors of alcohol dehydrogenase and aldehyde dehydrogenase, it was shown that ethanol metabolism was required for these changes in microtubules and that acetaldehyde most likely mediated these changes (Kannarkat et al. 2006). These alterations in microtubule dynamics could alter their ability to facilitate protein movement and could be one of the mechanisms responsible for the impairment in protein trafficking observed in alcoholic liver disease.

Researchers have used genetically engineered hepatoma-based cell lines that express either alcohol dehydrogenase, cytochrome P450 2E1, or both of these enzymes (VA-13, E47, and VL-17A cells, respectively) to study the effects of ethanol metabolism on proteasome function (Dai et al. 1993; Clemens et al. 2002; Donohue et al. 2006). The proteasome is a cellular organelle that, when signaled by the protein ubiquitin, is responsible for the majority of intracellular protein degradation. Culturing VA-13 and VL-17A cells in the presence of 25 mM ethanol (slightly higher than legal intoxication) for 3 days resulted in decreased cellular replication and increased cell death but had no effect on proteasome activity. Conversely, when these studies were repeated using higher concentrations of ethanol, decreased proteasome activity was observed in VL-17A cells, which express both alcohol dehydrogenase and cytochrome P450 2E1. This led to the suggestion that increased levels of reactive metabolites (i.e., reactive oxygen and nitrogen species) were generated in VL-17A cells cultured in the presence of higher concentrations of ethanol and that increased levels of reactive metabolites were required for ethanol-induced proteasome dysfunction. Additionally, using these three recombinant cell lines, it was found that treatment with interferon gamma (IFN-γ), an important protein involved in the immune response, activated the proteasome in VL-17A cells and that this increase in proteasome activity was inhibited by ethanol metabolism (Osna et al. 2003). Further investigations revealed that ethanol metabolism blunted the IFN-γ –induced activation of signal transducer and activator of transcription-1 (STAT-1), an important mediator of IFN-γ signaling. In addition, researchers found that impaired STAT-1 activation and proteasome activity was mediated, at least in part, by reactive oxygen and reactive nitrogen species generated by ethanol metabolism (Osna et al. 2005). These findings may help explain the increased susceptibility of alcoholics to some hepatic pathogens.

## Toxic Effects of Ethanol Metabolism on Hepatocyte Viability

Hepatic cells genetically engineered to metabolize ethanol have been extremely valuable tools in investigating the mechanisms by which ethanol damages hepatic cells. Using these cells, one of the first things that became evident was that ethanol itself is not normally toxic to hepatic cells, even at concentrations much higher than those normally detected in the blood of human beings consuming alcohol.

Because ethanol itself does not normally cause hepatic cellular toxicity, studies have focused on the toxic effects of ethanol metabolism. The development of the intragastric model of ethanol administration, in which ethanol is directly pumped into the stomach of experimental animals, revealed that enhanced cytochrome P450 2E1 activity was associated with more severe liver disease, implicating cytochrome P450 2E1 activity as a mediator of cell damage.

Cytochrome P450 2E1 activity is associated with the production of reactive oxygen species. To more closely investigate the mechanisms by which cytochrome P450 2E1 activation and the production of reactive oxygen species cause cellular damage, Dai and colleagues (1993) and Chen and Cederbaum (1998) developed recombinant hepatoma-based cell lines that express cytochrome P450 2E1; they called these cell lines E9 and E47 cells (Dai et al. 1993; Chen and Cederbaum 1998). Using these cells, they demonstrated that cytochrome P450 2E1–mediated ethanol metabolism resulted in cell damage (i.e., cytotoxicity). The severity of the cytotoxicity was time and dose dependent and was ameliorated by chemically inhibiting cytochrome P450 2E1 activity. Additionally, it was shown that the toxic effects associated with cytochrome P450 2E1–mediated ethanol metabolism could be inhibited by treating the cells with free-radical scavengers or antioxidants, implicating the production of reactive oxygen species as mediators of cell death. Expression of cytochrome P450 2E1 in cultured cells is associated with increased expression of the cellular free-radical scavenger glutathione, presumably to protect the cells from increased basal levels of reactive oxygen. Depletion of glutathione in these cells increased the toxic effects of ethanol, providing further evidence that cytochrome P450 2E1 and the production of reactive oxygen species are involved in ethanol metabolism–mediated cellular toxicity (Wu and Cederbaum 2003).

Free radicals also may have a role in the scarring of the liver (i.e., fibrotic response). Using cocultures of E47 cells and the star-shaped liver cells involved in the development of fibrosis (hepatic stellate cells), Nieto and colleagues (2002) showed that the production of reactive oxygen species increased a number of indices associated with activation of stellate cells, including the increased production of collagen type I, a major component of the fibrotic scarring associated with alcoholic liver disease.

It has been proposed that ethanol alone is not sufficient to cause liver disease but instead sensitizes the liver to the effects of other agents that normally are not toxic to hepatocytes. One of the agents to which ethanol metabolism is thought to sensitize the liver is tumor necrosis factor alpha (TNF-α). TNF-α is a protein involved in the immune response (i.e., cytokine) that is produced by activated Kupffer cells of the liver and is normally not toxic to hepatocytes; in fact, TNF-α is required for efficient liver regeneration. Pastorino and colleagues (2003) used Hep G2 cells and the cytochrome P450 2E1–expressing Hep G2 derivatives, E47 cells, to investigate the interaction between ethanol and TNF-α. They found that ethanol treatment sensitized Hep G2 cells to TNF-α–induced cell death. This TNF-α–induced cytotoxicity was triggered by activation of chemical signals leading to induction of programmed cell death (i.e., apoptotsis) and was mediated by the development of mitochondrial damage. In E47 cells, the toxic actions of ethanol and TNF-α occurred at lower ethanol concentrations and were more severe. Further studies showed that TNF-α–induced cytotoxicity was mediated by the sustained activation of the cellular signaling chemical, p38 MAPK, a member of the mitogen-activated protein kinase family. The sustained activation of p38 MAPK resulted in translocation of the proapoptotic protein BAX to the mitochondria, where it induced mitochondrial damage, resulting in the release of deleterious mitochondrial components and initiation of the apoptotic death pathway (Pastorino et al. 2003).

One of the first pathologic changes to the liver, in response to ethanol consumption, is accumulation of fatty acids and triglycerides, a condition known as fatty liver. Fatty liver once was thought to be a benign change to the liver but is now recognized as a precursor to a liver condition known as nonalcoholic steatohepatitis (NASH). Because of this, the alcohol-associated accumulation of fat in the liver has received considerable attention. Cultured cells have been used very effectively to help unravel the mechanisms by which fat accumulates in the liver as a result of ethanol metabolism. As mentioned above, ethanol metabolism results in many biochemical changes that occur simultaneously in the liver. Of these changes, the increase in the NADH/NAD^+^ ratio and the production of reactive oxygen species have drawn particular attention as mediators of fatty liver. Using HeLa^2^ cells that expressed alcohol dehydrogenase, Galli and colleagues (1999) investigated the contribution of these changes to the accumulation of fat. They demonstrated that alcohol dehydrogenase–mediated metabolism of ethanol was sufficient to cause the accumulation of lipids and that the increase in the NADH/NAD^+^ ratio was primarily responsible (Galli et al. 1999). Although the increased NADH/NAD^+^ ratio was sufficient to cause this increase in lipids, the authors suggested that other factors might be required to sustain the increased levels. In a subsequent study, these authors used hepatoma cells that expressed alcohol dehydrogenase to demonstrate that acetaldehyde reduced the activity of a nuclear receptor, peroxisome proliferator–activated receptor alpha (PPARα). The reduction in PPARα activity resulted in the accumulation of fat in the liver because PPARα normally activates many genes that are involved in the metabolism of fatty acids (Galli et al. 2001). These findings provided another mechanism by which ethanol metabolism alters normal hepatic metabolism of fat.

Cultured hepatoma cells also have been used to investigate the liver-damaging effects of nonoxidative metabolism of ethanol. Nonoxidative ethanol metabolism is mediated by a group of enzymes known as fatty acid ethyl ester synthases. Using parental and recombinant Hep G2 cells, Wu and colleagues (2006) investigated the involvement of nonoxidative ethanol metabolism in the presence and absence of oxidative ethanol metabolism. They found that fatty acid ethyl ester synthesis was increased in cells that did not express alcohol dehydrogenase and that the increased accumulation of fatty acid ethyl esters was associated with increased apoptotic cell death. The authors suggested that this finding could explain the mechanism of liver injury in the later stages of alcoholic liver disease, when alcohol dehydrogenase activity is diminished.

## Effects of Ethanol Metabolism on Cellular Replication

The liver has a tremendous capacity to replace cells that are lost or damaged by cytotoxic injury. In fact, if part of the liver is surgically removed, the remaining portion of the liver will increase in size to replace the mass of the original organ. Many animal studies, most of which used a model known as partial hepatectomy, in which a portion of the liver is surgically removed, have demonstrated that ethanol impaired the regenerative capacity of the liver. But as with most of the effects of ethanol on the liver, it was not initially clear whether ethanol or ethanol metabolism was responsible for this impairment.

Initial studies investigating this ethanol-induced impairment of cellular replication showed that culturing isolated hepatocytes in the presence of ethanol blunted their ability to replicate when stimulated with hormones or growth factors. Subsequent studies revealed that inhibiting ethanol metabolism partially abolished this dysfunction, implicating the requirement for ethanol metabolism in this ethanol-induced impairment. These findings were expanded using a hepatoma cell line. Culturing these hepatoma cells in the presence of ethanol not only impaired DNA synthesis but also resulted in a reduction in the cell density of the cultures. It was shown that ethanol metabolism slowed the cellular doubling time and increased the fraction of cells in the G1 phase of the cell cycle;^3^ thus impairing progression of cells through the cell cycle and inhibiting cellular replication. Additionally, these impairments were reproduced when cells were cultured in the presence of the ethanol metabolism byproduct acetaldehyde (Higgins and Borenfreund 1986).

To more closely investigate the role of acetaldehyde in these ethanol-induced impairments, we developed the VA13 cells. These Hep G2–based cells expressed alcohol dehydrogenase and produced acetaldehyde when cultured in the presence of ethanol. Initial studies showed that culturing VA-13 cells in the presence of ethanol resulted in a reduction in cell accumulation; this reduction resulted not only from cell death but also from impaired DNA synthesis and cellular replication. Additionally, these effects were not observed when VA-13 cells were cultured in the presence of alcohol used as rubbing alcohol (i.e., isopropanol). Metabolism of isopropanol results in most of the biochemical changes associated with ethanol metabolism but does not result in the production of acetaldehyde. Therefore, the fact that isopropanol metabolism had no effect on cell accumulation demonstrated the role of acetaldehyde in these impairments (Clemens et al. 2002).

Closer investigation of this ethanol-induced impairment in cellular replication revealed that culturing VA-13 cells in the presence of ethanol dramatically increased the number of cells in the G2/M transition^4^ of the cell cycle. An enzyme known as cyclin-dependent kinase 1 (CDK1) is required for cells to traverse the G2/M transition and undergo mitosis. Researchers found that culturing VA-13 cells in the presence of ethanol decreased the activity of CDK1, thus providing an explanation for the increase in the number of cells at the G2/M transition and the decrease in cellular replication (Clemens et al. 2003).

## Conclusions and Future Directions

Cultured cells have been successfully used to investigate a wide variety of ethanol-induced effects on the liver. From these studies, it is clear that the use of cultured cells has added to our understanding of the molecular mechanisms by which ethanol metabolism damages hepatic cells and ultimately causes liver disease. Use of these systems has allowed detailed investigation of the effects of individual metabolic pathways of ethanol metabolism and specific biochemical changes. Additionally, studies using cultured cells have allowed researchers to determine the contribution of these metabolic pathways and biochemical changes to specific ethanol-induced cellular impairments.

Although the use of cultured cells has many advantages, their use is not without limitations; the strength of these systems also is their weakness. Many immortalized cells lose the ability to express some enzymatic pathways; therefore, the specific cell line used must be carefully chosen. Use of cultured cells allows detailed studies of specific cellular functions but cannot take into consideration the interactions of the different cell types of the liver or the influence of the three-dimensional environment and extracellular matrix. Therefore, in the future, cocultures and cells suspended in three-dimensional matrices will be used to more closely mimic the liver. Additionally, culture systems in which the activity of cytochrome P450 2E1 is inducible have been developed (Huan et al. 2004; Schattenberg et al. 2005). Although these cell lines have not yet been used to study the effects of ethanol metabolism, use of cells in which the expression of ethanol-metabolizing enzymes can be induced or regulated will provide added versatility and may be extremely useful in furthering our understanding of the mechanisms by which ethanol metabolism alters cellular biochemistry and intracellular signaling. These modifications will provide additional insight into the mechanisms by which ethanol metabolism damages the liver and causes liver disease.

## Figures and Tables

**Figure:**
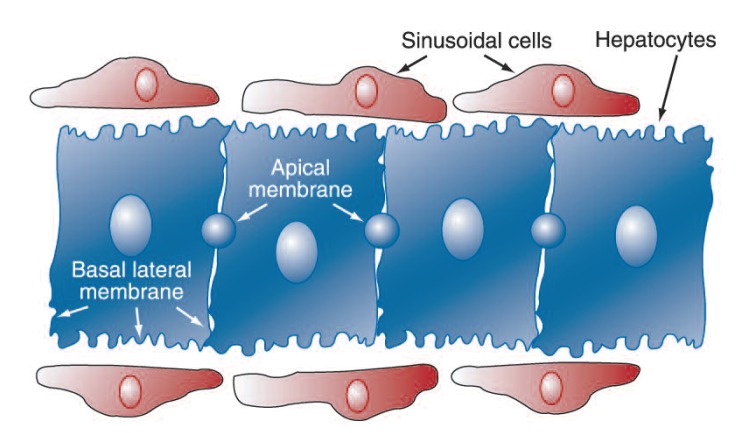
Schematic representation of the domains of the hepatocyte membrane. The cell membrane of a liver cell (i.e., hepatocyte), like other cells, is not uniform (i.e., exhibits membrane polarity). Different domains of the cell membrane have different functions. The basal lateral domain of the membrane is exposed to the liver sinusoid, or the blood supply, as well as the narrow intercellular space between adjacent hepatocytes, and the apical domain of the membrane is exposed to the tube or space between liver cells that collects bile from the cell (i.e., the bile canaliculus).

## Footnotes

1 Cells produce NAD from niacin and use it to transport electrons in redox reactions, in which atoms either gain electrons (reduction) or lose electrons (oxidation). During this process NAD gains a pair of electrons and a proton and thus is reduced to NADH, releasing one proton (H^+^).

2 HeLa cells are an established cell line used in medical research. The cell line was derived from cervical cancer cells taken from Henrietta Lacks, who died from her cancer in 1951.

3 The cell cycle is the series of events that must occur for a cell to replicate itself. During the G1 phase of the cell cycle cells increase in size, produce RNA, and synthesize protein.

4 The G2/M transition in the cell cycle is the progression from the G1 phase, after the completion of DNA synthesis, during which the cell grows and produces new proteins, to the M phase, during which the cell divides.
